# Supplementary figures and images for: The Comprehensive Analysis of m6A-Associated Anoikis Genes in Low-Grade Gliomas
Source: Brain Sci. 2023 Sep 12;13(9):1311. doi: 10.3390/brainsci13091311 (PMC10527396; doi:10.3390/brainsci13091311)

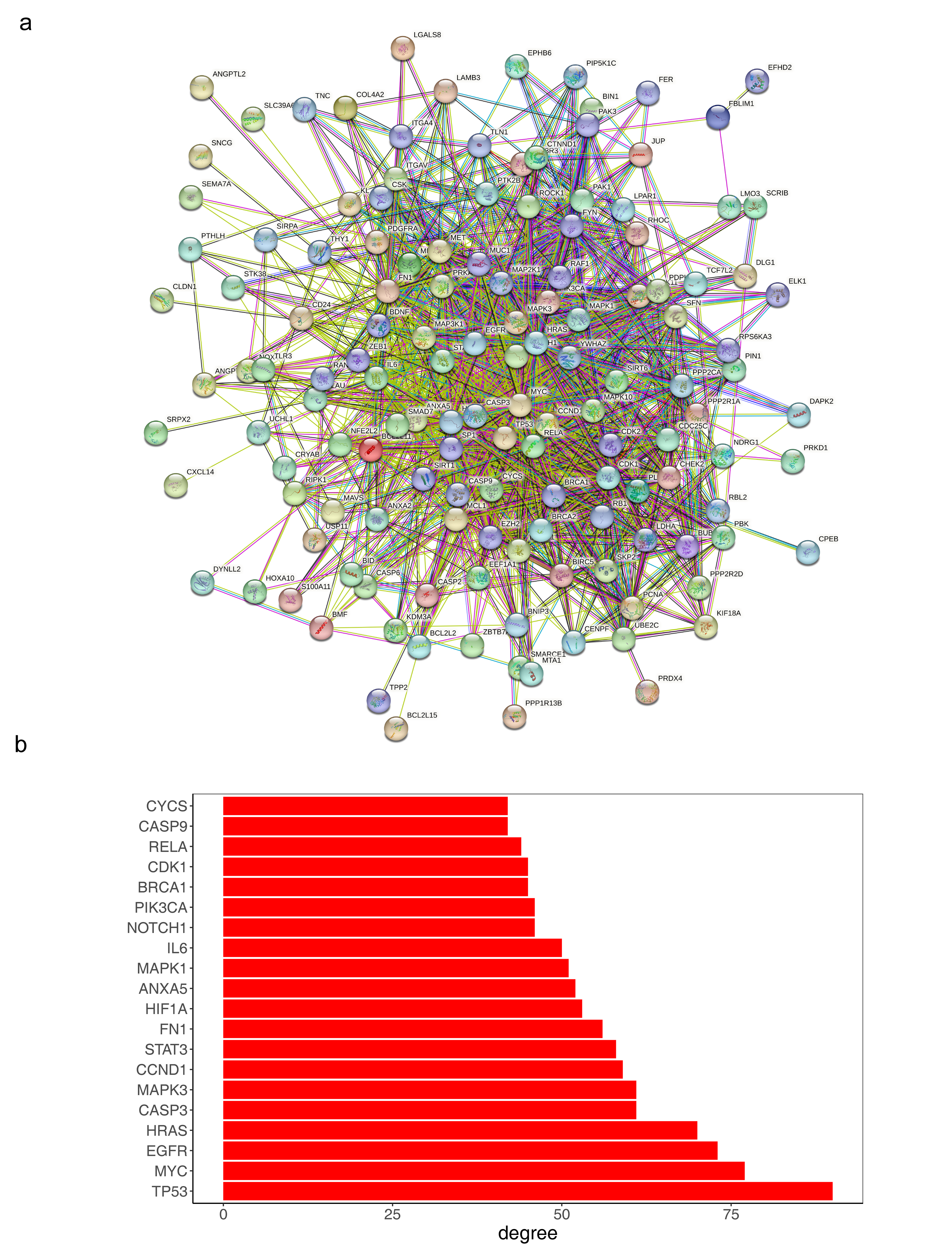

Supplement: Supplementary file 1 [file brainsci-13-01311-s001.zip › Figure S1.tif]

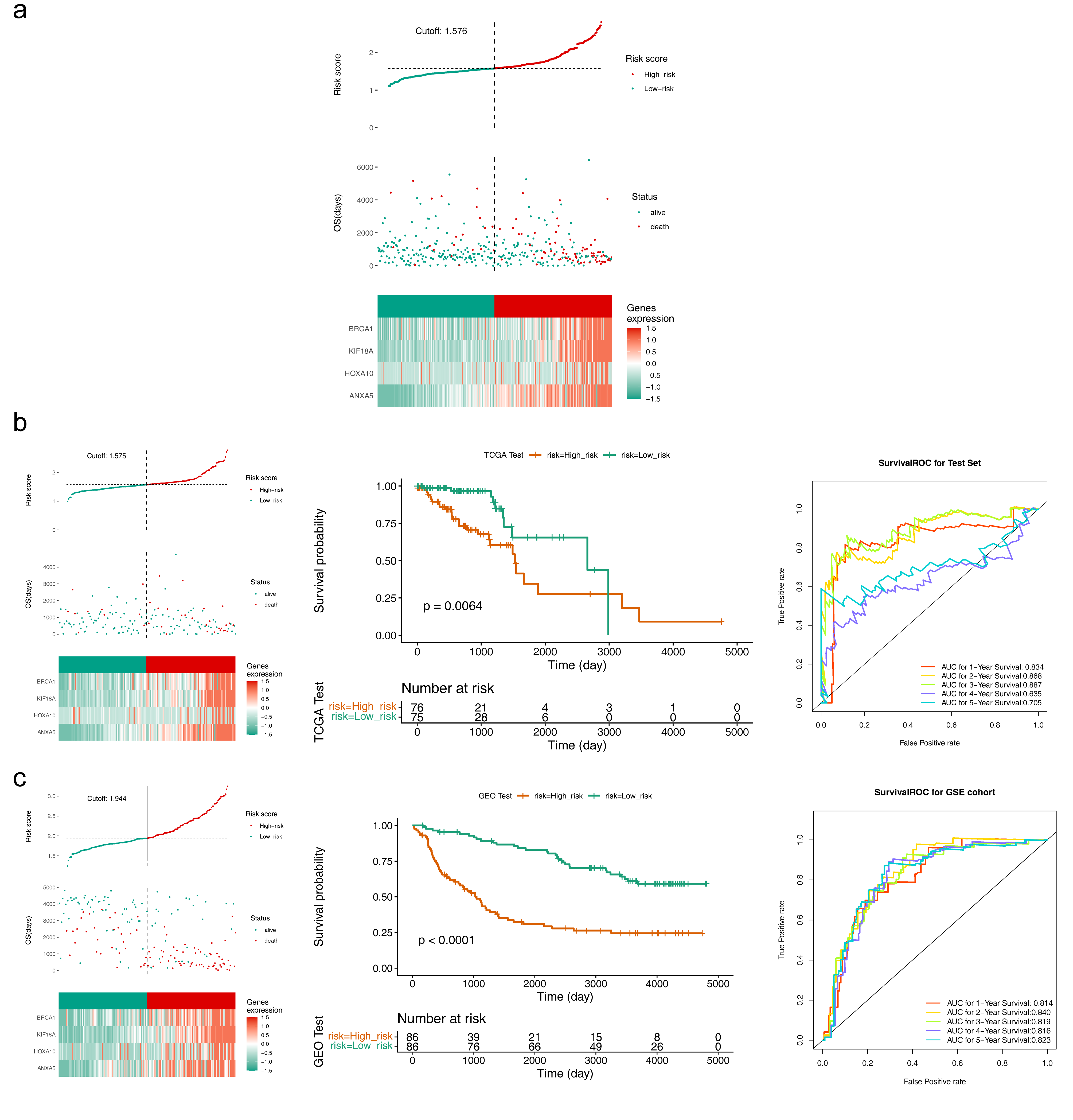

Supplement: Supplementary file 1 [file brainsci-13-01311-s001.zip › Figure S2.tif]

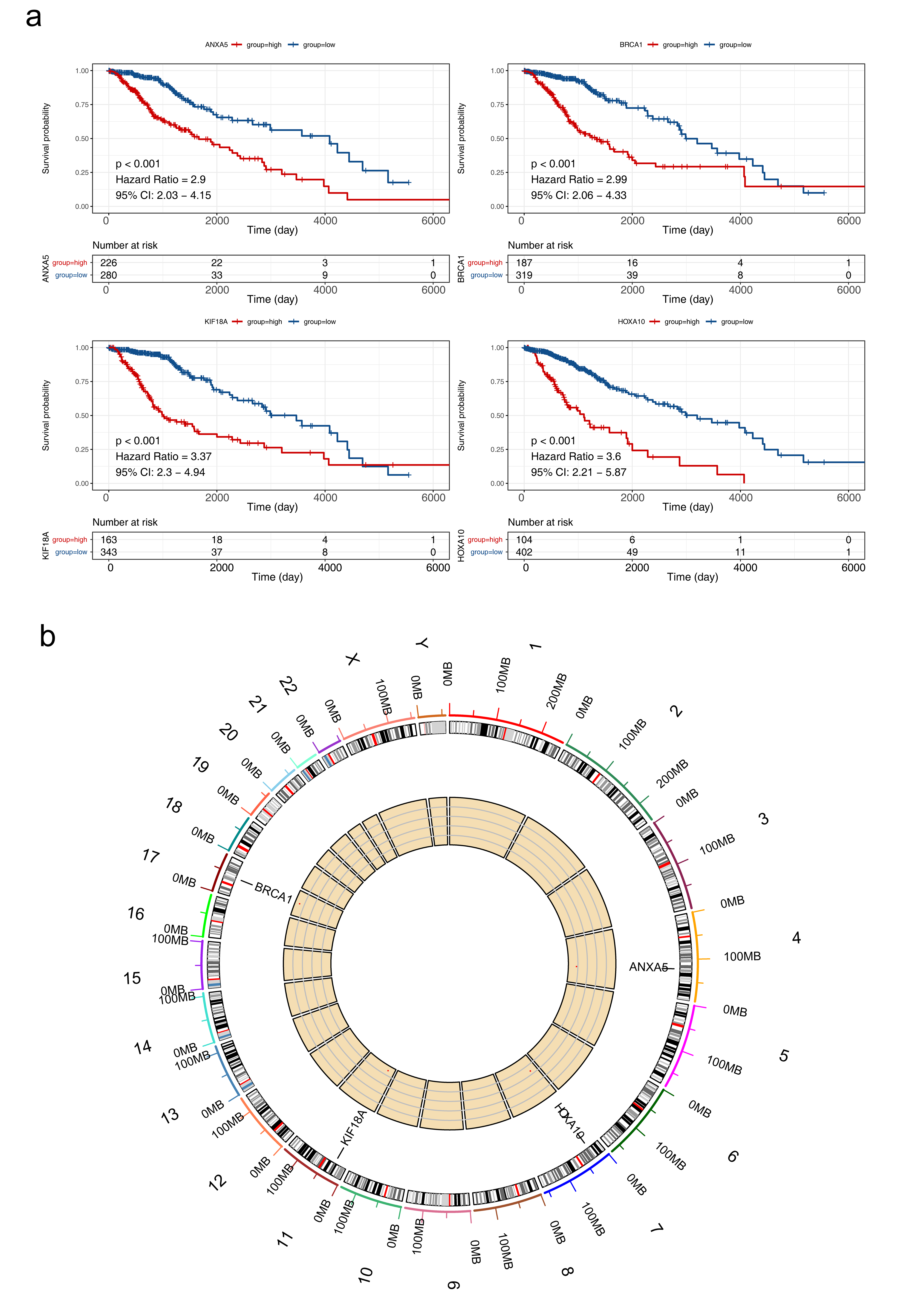

Supplement: Supplementary file 1 [file brainsci-13-01311-s001.zip › Figure S3.tif]

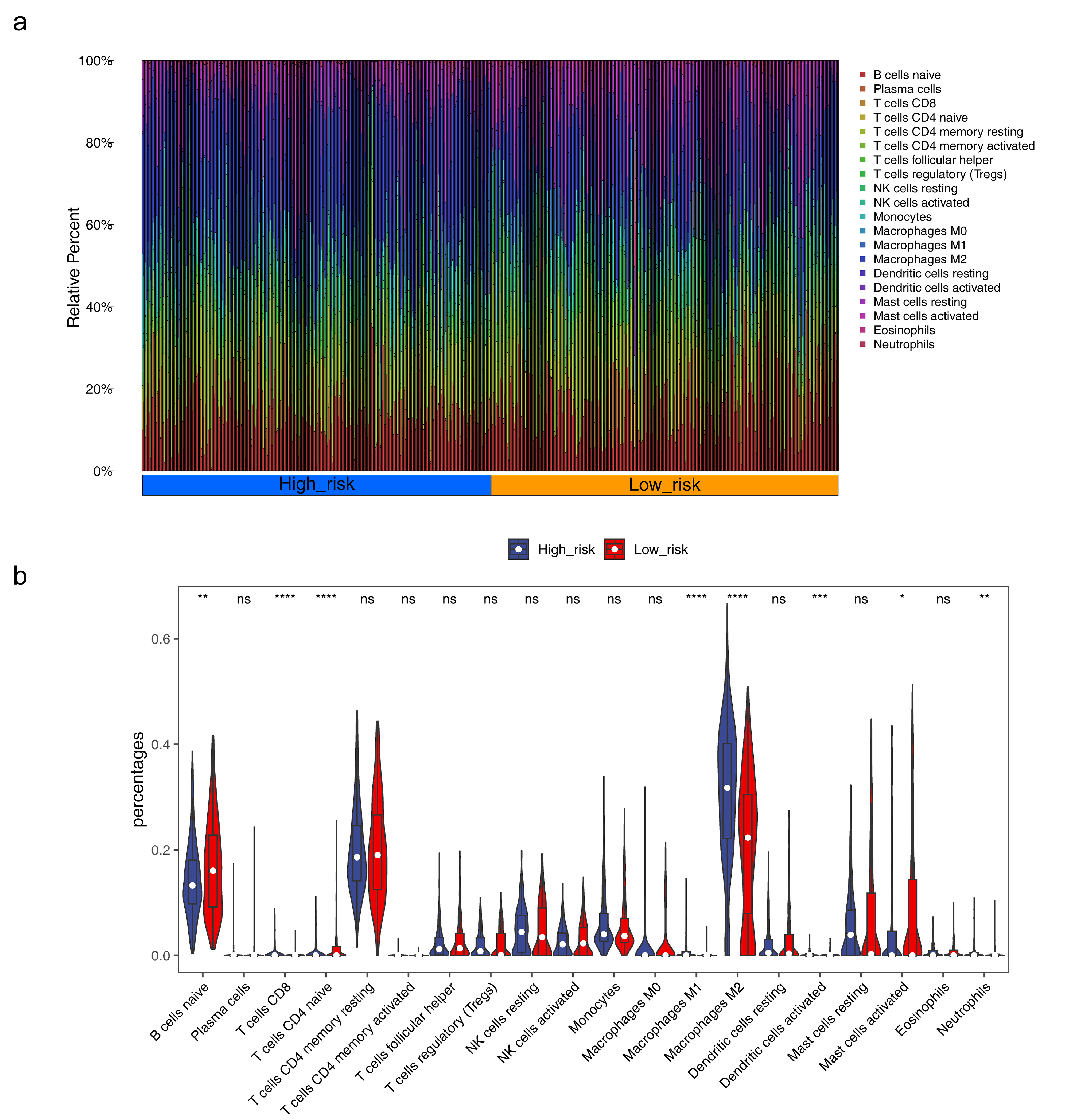

Supplement: Supplementary file 1 [file brainsci-13-01311-s001.zip › Figure S4.tif]

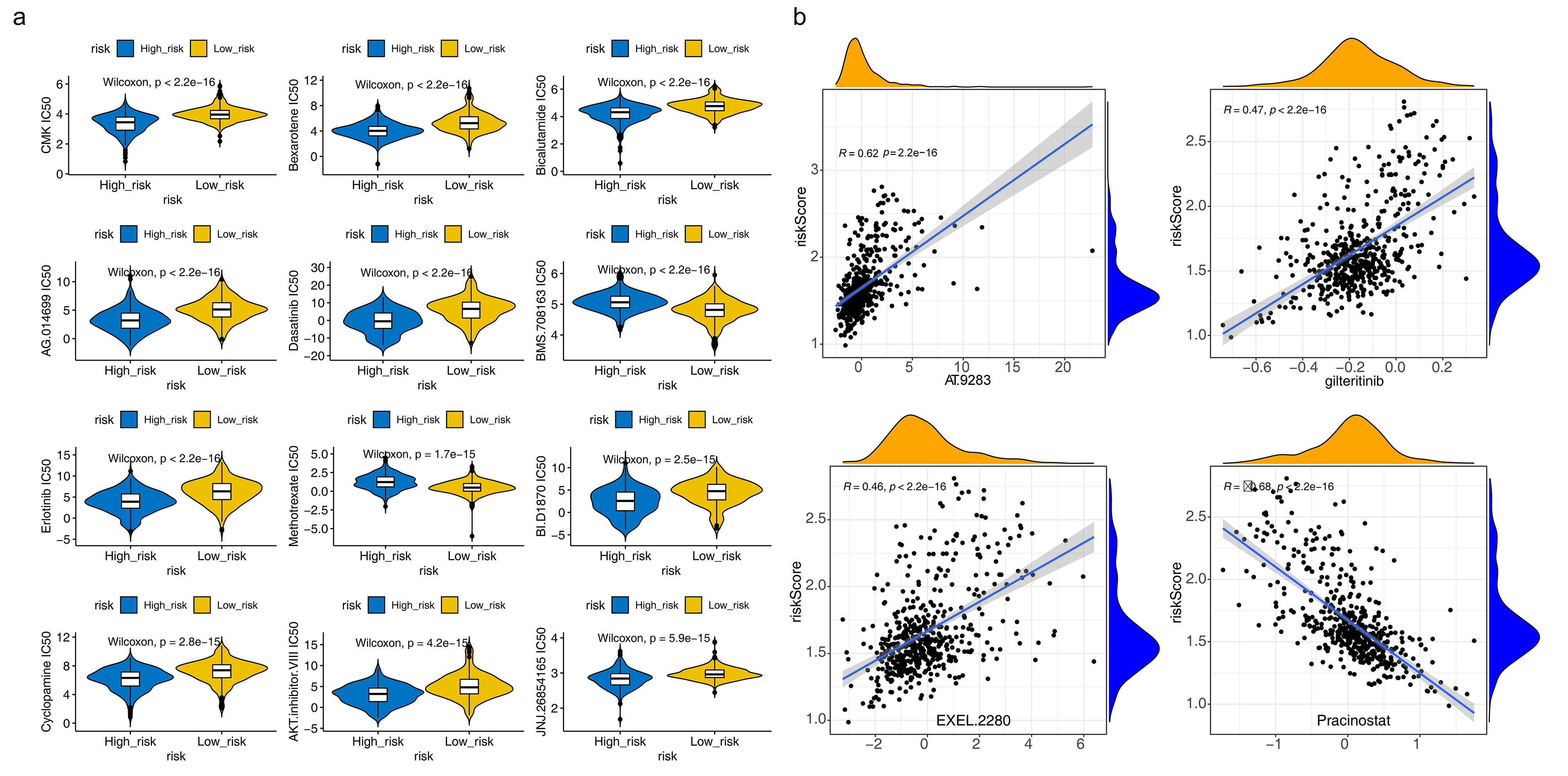

Supplement: Supplementary file 1 [file brainsci-13-01311-s001.zip › Figure S5.tif]
